# How Can We Personalize the Delivery of Onabotulinumtoxin‐A for Patients With Neurogenic Lower Urinary Tract Dysfunction?

**DOI:** 10.1002/nau.70290

**Published:** 2026-04-08

**Authors:** Shirley L. Wang, William I. Jaffe, Ariana L. Smith

**Affiliations:** ^1^ Division of Urology University of Pennsylvania Philadelphia Pennsylvania USA

**Keywords:** botulinum toxin, neurogenic detrusor overactivity, neurogenic lower urinary tract dysfunction, onabotulinum‐A, personalized treatment

## Abstract

**Background & Aims:**

Neurogenic lower urinary tract dysfunction (NLUTD) can produce bothersome urinary symptoms, impact quality of life, and in some cases, lead to deterioration of upper urinary tract function. Intradetrusor injection of onabotulinumtoxin‐A (BoNT‐A) is approved for NLUTD in patients who have an inadequate response to or intolerance of oral pharmacologic therapy (with beta‐3 agonists and/or antimuscarinics) for urinary frequency, urgency, urgency incontinence, and/or impaired compliance. Modifications of its use, including dose, injection location, number of injections, setting of procedure, combination therapy with oral agents, and use among different patient populations have since been explored. Here, we aim to review the literature on BoNT‐A in NLUTD with a focus on unique neurologic patient groups as well as patient and technical factors to inform personalized treatment strategies.

**Methods:**

We performed a narrative review evaluating the existing literature on BoNT‐A in NLUTD, focusing on outcomes across neurologic etiologies, and variation in patient‐specific and technical factors.

**Results:**

Across neurologic etiologies, BoNT‐A improves quality of life and urodynamic parameters with low rates of adverse effects. Patient factors, including baseline voiding status, ability to self‐catheterize, and age are important considerations in treatment selection and initial dosing of BoNT‐A. Technical modifications such as reduced injection sites or trigone‐including templates have been suggested to reduce adverse effects or improve efficacy but require further study.

**Conclusions:**

BoNT‐A is an established therapy for NLUTD. Individualized treatment strategies that account for patients’ neurologic etiology and functional status are beneficial in optimizing outcomes.

## 1 Introduction

A breadth of neurologic conditions can cause neurogenic lower urinary tract dysfunction (NLUTD), including multiple sclerosis (MS), spinal cord injury (SCI), cerebrovascular accident (CVA), and Parkinson's disease (PD). If untreated or poorly managed, NLUTD may result in recurrent urinary tract infections or deterioration of upper tract function. NLUTD refers to abnormal function of the bladder, bladder neck, or urethral sphincters and can cause significant bothersome symptoms, including urinary frequency, urgency, incontinence, and difficulty emptying the bladder. Initial treatment typically consists of pharmacologic therapy aimed at the most bothersome symptoms and may include combination therapy aimed at both bladder storage and bladder emptying symptoms [1].

Onabotulinumtoxin‐A (BoNT‐A) emerged as an alternative for those who fail pharmacologic therapy with persistent urinary frequency, urgency, urgency incontinence, and/or impaired compliance. BoNT‐A is a neurotoxin derived from the organism *Clostridium botulinum* [2]. BoNT‐A cleaves synaptosome‐associated protein (SNAP‐25) at the neuromuscular junction, inhibiting release of acetylcholine and causing paralysis of the detrusor muscle [2]. Evidence additionally supports an inhibitory effect on afferent pathways, correlating to an improvement in sensation of urgency [2]. BoNT‐A was approved by the FDA in 2011 for neurogenic detrusor overactivity (NDO) causing incontinence in patients who fail or cannot tolerate anticholinergic medications (prior to release of beta‐3 adrenergic agonists). The starting dose recommended by the FDA for NDO is 200U rather than the recommended dose of 100U for idiopathic overactive bladder (OAB) [3].

While existing literature has evaluated the use of intradetrusor BoNT‐A in a range of neurologic conditions, guidance for the personalization of specific neurologic populations and clinical scenarios is limited. This narrative review, therefore, aims to provide a clinically focused synthesis of the available evidence as well as patient‐ and procedure‐related factors that inform individualized treatment strategies for patients undergoing BoNT‐A injections.

### 1.1 Multiple Sclerosis

Multiple sclerosis (MS) is an immunological disorder resulting in neuronal loss and demyelination. Up to 90% of patients with MS experience bladder dysfunction. The majority of patients have cervical lesions and experience neurogenic detrusor overactivity (NDO) [4]. The use of BoNT‐A for NDO secondary to MS is supported by findings from the Double‐blind InvestiGation of purified Neurotoxin complex In neurogenic deTrusor overactivitY (DIGNITY) trials, which included two double‐blind, placebo‐controlled phase 3 studies [1, 5, 6]. These studies enrolled patients with MS or SCI with at least 14 episodes of urgency incontinence per week who had failed a trial of anticholinergics due to ineffectiveness or intolerance of side effects [6]. A total 381 patients with MS were included. At 6 weeks, patients had significant improvement in urgency incontinence episodes per week (mean reduction of 22.6 episodes per week), quality of life, and urodynamic parameters, including maximum cystometric capacity and maximum detrusor pressure at first involuntary detrusor contraction [6]. These studies showed no greater benefit among the group receiving 300U BoNT‐A, with a higher rate of urinary retention requiring initiation of clean intermittent catheterization (CIC) in the group receiving 300U versus 200U (47.1% vs. 31.4%) [6].

In patients with MS, it is important to balance treatment of the storage symptoms with the potential for worsening emptying symptoms. This may be accomplished with the use of lower doses in individuals who are spontaneously voiding. A randomized, double‐blind trial demonstrated efficacy of 100U BoNT‐A compared to placebo in reducing incontinence and improving urodynamic parameters and quality of life in patients with MS [7]. The rate of initiating CIC among patients receiving 100U BoNT‐A was 15.2% in this trial, compared to 31.4% among the patients who received 200U in the DIGNITY trials [6].

### 1.2 Spinal Cord Injury

Urinary dysfunction occurs in up to 80% of patients with spinal cord injury [8]. The location of the spinal cord injury impacts the type of dysfunction, with supra‐sacral injuries typically result in detrusor overactivity and detrusor striated sphincter dyssynergia (DESD). Dis‐coordinated contraction lead to high voiding pressure, incomplete bladder emptying, with potential for upper tract deterioration and renal failure, whereas sacral spinal cord injuries are more likely to cause acontractile bladder with possibility of poor compliance [8].

The use of BoNT‐A for NDO secondary to spinal cord injury was first described by Schurch et al. in 2000 [9]. The DIGNITY trials included 310 patients with SCI, 84.8% of whom used CIC at baseline [6]. Similar efficacy was observed between the groups receiving 200U versus 300U in improving clinical, quality of life, and urodynamic measures, with similar adverse event profile [5]. As only 15% of patients were spontaneously voiding, new‐onset retention was not compared between the two cohorts. A placebo‐controlled dose‐response study compared administration of 50U, 100U, or 200U in patients with SCI. Only the cohort receiving 200U had significant improvement in incontinence, with a similar rate of adverse events across all doses [10].

#### 1.2.1 Autonomic Dysreflexia

Patients with spinal cord injury at or above the level of T6 may develop autonomic dysreflexia (AD), a condition characterized by an exaggerated autonomic reaction in response to stimulus below the level of the injury [11]. Care should be taken during cystoscopic procedures to avoid overfilling. Cardiovascular monitoring should be performed if these patients are undergoing procedures in the office setting, with immediate cessation if the patient develops hypertension or symptoms including flushing, diaphoresis, or anxiety [1]. It is important to rapidly decompress the bladder and bring the patient to an upright position if symptoms occur. For persistent symptoms, application of 1 to 2 inches of 2% nitroglycerine paste on the skin, above the level of the spinal cord lesion is needed. This medication should be readily available in the procedure area. Several studies have observed improvement in self‐reported symptoms and objective measures of AD after intradetrusor BoNT‐A injection [9, 12].

#### 1.2.2 Detrusor External Sphincter Dyssynergia

DESD, or uncoordinated contraction of the detrusor and external urinary sphincter, occurs due to suprasacral lesions of any etiology and is most commonly seen among patients with SCI and MS [4]. DESD results in incomplete emptying and elevated intravesical pressure. Intradetrusor BoNT‐A, combined with CIC, may help patients achieve continence with acceptable detrusor pressures.

Intrasphincteric BoNT‐A has been proposed as an alternative to surgical sphincterotomy. A meta‐analysis of studies evaluating the use of 100U to 150U BoNT‐A injections to the external sphincter either transperineally or endoscopically showed significant decrease in mean detrusor pressure (88.7 to 20.5 cm H_2_O) [13]. Across all studies, PVR decreased by about 100 mL, and a small number of patients were able to discontinue CIC, although a few patients had worsening urgency and incontinence [13]. As intrasphincteric BoNT‐A targets skeletal muscle of the urethral sphincter, patients may require more frequent injections than with intravesical injections into smooth muscle (3–4 months vs. 6‐9 months) [13].

### 1.3 Cerebral Palsy

Cerebral palsy (CP) is a non‐progressive neurological disorder impacting motor function and posture. Over half of adults with cerebral palsy report lower urinary tract symptoms, with one study reporting overactivity and dyssynergic voiding in 30% and 12% of patients, respectively [14]. As DESD results from a spinal cord abnormality, whereas dyssynergic voiding in the setting of CP is due to a cortical lesion, this pattern of voiding in this population is referred to as pseudo‐detrusor sphincter dyssynergia (PDSD). Few studies have evaluated the use of BoNT‐A in this population: one series of 24 CP patients highlighted efficacy of BoNT‐A in detrusor overactivity, with 29.2% of patients continuing injections at median 4 years of follow‐up, and 16.7% discontinuing due to adverse events, although rates of UTI or retention were not reported [15]. Prior studies have described injection of BoNT‐A in the external urethral sphincter for PDSD in patients with CP, with improvement in urinary retention (65%), urinary frequency (48%), and reduction of UTIs (22%) with 60% of patients receiving repeat injections at follow‐up [16]. Evidence to support the use of BoNT‐A in adults with CP is limited, with initial series suggesting benefit for detrusor overactivity and PDSD.

### 1.4 Parkinson's Disease

Parkinson's Disease is a progressive disease characterized by distinct motor symptoms, cognitive dysfunction, and autonomic dysfunction [17]. Lower urinary tract symptoms affect 27%–85% of patients with PD [17]. BoNT‐A may be of particular interest in patients with PD given cognitive side effects associated with antimuscarinics [18] and financial toxicity of beta‐3 adrenergic agonists [19]. The first reported use of BoNT‐A in this population noted improvement in urinary frequency, urgency, quality of life, and urodynamic parameters with 200U BoNT‐A [20]. In this series, mean post‐void residual (PVR) rose after BoNT‐A injection, although no patients required initiation of CIC. Follow‐up studies have demonstrated similar efficacy of a reduced dose (100U), similarly without new instances of self‐catheterization [21, 22].

### 1.5 Cerebrovascular Accident

Stroke, or cerebrovascular accident (CVA), is a common neurologic condition causing chronic lower urinary tract symptoms in up to 19% of affected patients, with prevalence ranging depending on the location of the lesion [4]. While detrusor areflexia can often result in the acute period following CVA, detrusor overactivity is commonly seen in the chronic recovery phase [4]. Few studies have evaluated the use of BoNT‐A in this population. A retrospective analysis of 23 patients with CVA, nine with PD, and eight with dementia who received intradetrusor injection of 100U BoNT‐A showed comparable improvement in urgency and incontinence episodes, with similar increase in PVR as compared to the control group with idiopathic OAB [23]. Jiang et al. reported total continence in 30.6% of their 36 patients who received intradetrusor injection of 100U BoNT‐A, and reduction in incontinence episodes in another 55.6% of patients [24]. The rate of urinary retention in these patients is reported between 13.9% to 25% [23, 24].

Similar to patients with MS, patients with stroke may have other factors such as sex, body habitus, functional status, vision and manual dexterity that may affect their ability to CIC, and are important to consider when discussing the potential risks of BoNT‐A.

### 1.6 Patient Factors

#### 1.6.1 Voiding Status

Limited quality data exist on risk factors predictive of incomplete emptying or urinary retention after BoNT‐A injection, particularly among patients with NDO. Urodynamic testing may help to understand voiding dynamics such as the presence and strength of a detrusor contraction during voiding, the voiding efficiency, the presence of bladder outlet obstruction, and the PVR. While these parameters have not consistently predicted urinary retention in prior studies, taken together they can assist the clinician when counseling patients about the potential for risk of poor emptying or retention after BoNT‐A.

In those who are already on CIC, higher doses of BoNT‐A can be used without concern for urinary retention (Table 1). In patients who are volitionally voiding with elevated PVRs, BoNT‐A must be used with caution and careful follow‐up of voiding function post‐procedure. Generally, a dose of 100U is used, and assessment of emptying performed 1–2 weeks after injection. For patients with worsening emptying symptoms or function, initiation of CIC can alleviate these symptoms and allow recognition of improvement in storage symptoms. Some patients prefer this management and even opt for higher doses of BoNT‐A in the future. Others may opt to decrease the BoNT‐A dose or use alternative strategies for bladder management.

**Table 1:** Recommended starting dose for intradetrusor BoNT‐A injections in patients with NLUTD.

100 Units	200 Units	Consider alternative treatment
Patients volitionally voiding without catheter assistance Patients partially voiding with catheter assistance Patients with risk factors for retention (e.g. male, BPH, elderly) Patients in whom preservation of voiding is prioritized (e.g. MS, PD)	Patients reliant on intermittent catheterization Patients with indwelling catheter	Elevated PVR Unwilling or unable to self‐catheterize or accept indwelling catheter Patients with poor compliance and concern for upper tract deterioration

#### 1.6.2 Poor Compliance

The evidence to support use of BoNT‐A among patients with poor compliance is limited. A subgroup analysis of participants in the DIGNITY trials who had low baseline compliance (≤ 20 mL/cm H_2_O) reported improvement, defined as greater than 1 mL/cm H_2_O, in 93.4% of patients [25]. However, compliance was determined using maximum detrusor pressure at the first involuntary detrusor contraction rather than at the end of the filling phase, which may limit the clinical applicability of these findings. In a retrospective study including patients with baseline compliance ≤ 20 mL/cm H_2_O, improvement ≥ 5 mL/cm H_2_O was observed in 31% of patients after 300U BoNT‐A [26]. While the mean improvement in compliance among this subgroup was substantial (31.7 vs. 10.6 mL/cm H_2_O), no predictors of response were identified. Small case series have reported unchanged compliance among patients with low baseline compliance (29.26 vs. 28.76 mL/cm H_2_O) [27]. These findings likely reflect the fibrosis underlying poor compliance, which may be less responsive to BoNT‐A than detrusor overactivity. Patients with low compliance at baseline should be closely monitored for upper tract deterioration, particularly as bothersome urgency or incontinence may improve after BoNT‐A despite persistently elevated storage pressures. Ultimately, treatment with indwelling catheter drainage, augmentation cystoplasty, or urinary diversion may be required [1].

#### 1.6.3 Ability to Catheterize

Patient factors, including sex, functional status, cognition, vision, and body habitus, may limit patients’ ability to perform CIC and should be accounted for when considering BoNT‐A injection. Predictors of adherence to CIC include upper extremity dexterity, male gender, lower body mass index, and younger age [28]. Male and female anatomy represent different challenges to CIC, with men having a longer urethra to traverse and women having more difficult access to the urethra. If body habitus, functional status, and manual dexterity are suboptimal, CIC may not be possible without the assistance of a caregiver. While patients may be counseled that a lower dose confers a lower risk of urinary retention [6], complete unwillingness to have an intermittent or indwelling catheter is a contraindication to the procedure.

#### 1.6.4 Elderly Patients

BoNT‐A injection may be a more appropriate treatment option for elderly patients than anticholinergics to avoid deleterious effects on cognitive function. While beta‐3 agonists are safe in the elderly, insurance coverage and cost of these medications can be barriers to use, whereas BoNT‐A is generally covered by Medicare coverage plans. Physicians may be more hesitant to pursue procedural treatment in elderly patients due to increased frailty and comorbidities in this population. Elderly patients with idiopathic OAB are at higher risk of urinary retention after BoNT‐A injection [29]. A claims‐based analysis of nursing home residents with mean age 78 years showed treatment success, defined as repeat BoNT‐A injection within a year, of 34% [30]. The 30‐day complication rate was 38.4% and new urinary retention rate (presence of a new catheter in 3 months) 14.6% [30]. Patients aged ≥ 85 years were less likely to undergo repeat injection; however, frailty was not a predictor of treatment failure. Among this population in particular, providers should lead patient‐centered conversations on continuing with injections versus transitioning to an indwelling catheter or external collection device.

#### 1.6.5 Male Patients

Male anatomy may increase the risk of urinary retention after BoNT‐A and may be especially concerning in men with benign prostatic hyperplasia (BPH) and/or elevated PVR [31]. A retrospective review of 69 men receiving BoNT‐A, including 18 with NLUTD, found elevated PVR (≥ 50 mL) and dose greater than 100U to be predictive of urinary retention requiring catheterization [32]. Other studies provide conflicting evidence on the utility of pre‐operative bladder outlet obstructing index (BOOI) or PVR as predictors of post‐operative retention, although these are heterogeneous in inclusion criteria and outcomes [33]. Pre‐BoNT‐A urodynamics are generally recommended to aid in counseling regarding the risk of acute urinary retention after BoNT‐A injection.

### 1.7 Technical Considerations

#### 1.7.1 Anesthesia

BoNT‐A injections may be given in the office with local anesthetic in patients with bladder sensation. This spares the risk of intravenous anesthetic among patients who may have comorbidities, putting them at higher risk of anesthesia‐related complications. Use of a flexible cystoscope with compatible injection needles may be advantageous among patients with severe contractions who are unable to be placed in lithotomy position. The patient's ability to tolerate bladder filling (and risk of AD) should be considered when selecting sedation versus local anesthetic. For patients with preserved bladder sensation undergoing BoNT‐A injection in the office, instillation of an intravesical and intraurethral lidocaine has been demonstrated to provide satisfactory analgesia [34, 35].

#### 1.7.2 Number of Injections

Different injection numbers have been proposed as an alternative to the 30‐injection template used in the pivotal clinical trials. Avallone et al. prospectively evaluated the efficacy and safety of one to three injections of 100‐300U (each injection containing 100U) in 45 patients, half of whom had NDO [36]. They found a comparable rate of clinical improvement across injection numbers, with 52% of NDO patients achieving complete continence. They reported relatively low rate of symptomatic UTI (11.1%) and no episodes of urinary retention or systemic adverse events.

Other studies assessing a varied number of injections have excluded patients with NDO, though their results may be applicable to this population [37, 38]. These studies demonstrate similar efficacy and safety of reduced injections. Of note, DiCarlo‐Meachem et al. exclusively performed procedures in the clinic and showed patients who received fewer injections (5 vs. 20) were more willing to undergo a repeat procedure [38]. Fewer injections may be appropriate in the office setting to decrease procedure time and patient discomfort, without compromising efficacy. Decreased number of injections theoretically decreases the risk of UTI given fewer disruptions to the urothelium, although higher level evidence is required to support this.

#### 1.7.3 Injection Location

The package insert recommends intradetrusor BoNT‐A injections, avoiding the trigone [3]. In early studies and the pivotal randomized controlled trials, the trigone was spared due to theoretical concern for causing vesicoureteral reflux [5, 39]. Trigonal injections have since been demonstrated to be efficacious without causing reflux or greater risk of retention [39, 40, 41]. A randomized controlled trial including 36 patients with NDO showed superiority of a trigone‐including template in improving incontinence (80.9% decrease in incontinence vs. 52.44%, *p* < 0.001) [40]. Another randomized controlled trial including 91 patients with NDO due to SCI showed greater improvement in the trigone‐including group in quality of life, incontinence, and urodynamic parameters [41].

### 1.8 Concomitant Medical Therapy

The DIGNITY trials showed no additional improvement in urinary incontinence episodes, urodynamic parameters, or quality of life among patients who were taking an antimuscarinic in addition to BoNT‐A injection [6]. Continuation of antimuscarinics may still be considered for patients who had suboptimal response to these medications but tolerated their side effects.

There is increasing data to support the use of the beta‐3 adrenergic agonists mirabegron and vibegron for the medical management of NDO, and their use is now supported by the AUA NLUTD guidelines [1]. Few studies have investigated the use of beta‐3 adrenergic agonists in addition to BoNT‐A injection, and none to date in the NDO population. An open‐label prospective study compared the use of mirabegron or solifenacin with BoNT‐A to BoNT‐A alone in patients with idiopathic OAB [42]. The group receiving mirabegron with BoNT‐A had the greatest improvement in quality of life, incontinence episodes, and frequency.

Alpha blockers have been shown to be effective in increasing flow rate among patients with NLUTD [43]. Although alpha blockers are unlikely to resolve urinary retention secondary to NLUTD or BoNT‐A, their concomitant use may be beneficial among male patients who are able to spontaneously void. Further studies evaluating the concomitant use of oral pharmacotherapy alongside BoNT‐A injection are warranted.

### 1.9 Antibiotic Prophylaxis

Urinary tract infection (UTI) after BoNT‐A injection are reported in up to half of all patients, although definition of UTI (i.e. positive culture *vs.* symptomatic infection) is heterogeneous across studies [3]. The DIGNITY trials reported UTI rate up to 59%, without significant variation based on dosage; however, these trials did not distinguish between symptomatic versus asymptomatic UTIs [6]. A prospective study by Mouttalib et al. investigated the rate of UTI after BoNT‐A injection among patients who routinely self‐catheterize, who had negative pre‐operative urine cultures [44]. Patients did not receive peri‐procedure antibiotic prophylaxis. Within a week of the procedure, 38% of patients had a positive urine culture; of these, 19% had a symptomatic UTI, and 81% were asymptomatic. Thus, culture‐directed perioperative antibiotic prophylaxis should be provided, particularly for patients who self‐catheterize or have an indwelling catheter and have a greater likelihood of bacterial colonization of the urinary tract.

### 1.10 Follow‐Up and Repeat Dosing

Patients who are spontaneously voiding should have follow‐up within several weeks to ensure they are voiding with acceptable PVRs. Patients with impaired compliance and NLUTD who are newly continent after BoNT‐A injection should undergo repeat urodynamics to ensure their storage pressure remains within a safe range to prevent long‐term damage to the upper tracts. In the extension study of the DIGNITY trials, the median duration of effect was 9 months, with sustained improvement in urinary incontinence episodes at each subsequent injection, and no change in the profile of adverse events [45]. Several other studies have demonstrated safety and efficacy of repeated injections [45, 46, 47].

In an analysis of 140 patients with heterogenous etiologies of NLUTD, half discontinued use over a 10‐year period [48]. Of these patients, half discontinued due to primary or secondary lack of improvement. A retrospective study of MS patients reported 81% of patients continued to receive BoNT‐A injections after five treatments [49]. A score on the MS‐specific Expanded Disability Status Scale ≥ 6 (i.e. dependent on assistance for ambulation) and the progressive subtype of MS were predictive of greater likelihood of discontinuation of BoNT‐A. Inadequacy of BoNT‐A for management of NDO due to progression of MS or neurologic disorders may result due to worsening urinary symptoms or worsening musculoskeletal symptoms limiting patients' ability to CIC.

Physicians should counsel patients, particularly younger patients with progressive conditions, that BoNT‐A injections may not keep up with progression of their disease and therefore may not be a viable lifelong treatment for NDO. Further work may better elucidate mechanisms of secondary failure and barriers to adherence.

## 2 Conclusion

Onabotulinumtoxin‐A improves symptoms and urodynamic parameters in patients across the spectrum of NLUTD. Its efficacy and safety have been demonstrated in patients with neurogenic detrusor overactivity. Patients’ characteristics, including etiology and type of NLUTD, age, sex, vision, manual dexterity, and functional status should be considered when performing BoNT‐A injections. Further work may better clarify patient factors which predict response, adverse events, and impact of technical modifications which would enable physicians to better tailor this treatment to their patients.

## Author Contributions

Shirley L. Wang drafted and wrote the majority of the manuscript. William I. Jaffe and Ariana L. Smith provided critical review, revisions, and oversight of the content. All authors approved the final version of the manuscript.

## Funding

The authors have nothing to report.

## Ethics Statement

This review study was exempt from institutional review board review.

## Conflicts of Interest

The authors declare no conflicts of interest.

## Data Availability

Data availability statement, clinical trial registration, and patient consent statement not applicable to this narrative review. There are no materials reproduced from other sources.
